# Frequency and determinants of hypoferritinemia without anemia among reproductive age women in Lahore: A cross-sectional study

**DOI:** 10.12669/pjms.41.12.12789

**Published:** 2025-12

**Authors:** Shehla Javed Akram, Abdul Majeed Akhtar, Rubeena Zakar

**Affiliations:** 1 Shehla Javed Akram, MBBS, DTM & H, MPhil Public Health., University Institute of Public Health, Faculty of Allied Health Sciences, The University of Lahore, Lahore, Pakistan; 2 Abdul Majeed Akhtar, MBBS, PhD Public Health., University Institute of Public Health, Faculty of Allied Health Sciences, The University of Lahore, Lahore, Pakistan; 3 Rubeena Zakar, MBBS, PhD Public Health., Department of Public Health, University of the Punjab, Lahore, Pakistan

**Keywords:** Determinants, Dietary diversity, Females, Hypoferritinemia without anemia, Iron deficiency without anemia, Serum ferritin, Symptoms

## Abstract

**Objective:**

To determine the frequency and determinants of Hypoferritinemia without anemia (HWA) among women of reproductive age in Lahore, Pakistan.

**Methodology:**

This cross-sectional study was conducted in four tertiary-care hospitals and their adjoining areas. Using simple random sampling, 1,347 women between the ages of 18-45 were selected. Patients from hospital OPDs who met the eligibility criteria and provided informed consent were selected to participate in the study. A structured questionnaire was filled out for each participant and blood samples were collected for CBC and serum ferritin evaluation.

**Results:**

HWA was present in 399 (29.6%) of the participants. Lethargy/feeling of tiredness was the most common symptom (90.7%). Regression analysis showed that rural residence (AOR = 3.04, p = 0.016), unemployment (AOR = 3.54, p = 0.004), use of medication (AOR = 15.02, *p* < 0.001), heavy menstrual bleeding: including duration >7 days (AOR = 7.49, *p* < 0.001), and use of > 2 pads/day (AOR = 3.21, p = 0.040), low meal frequency (<2/day) (AOR = 9.45, *p* < 0.001), breakfast skipping (AOR = 4.67, p = 0.029), and low dietary diversity (AOR = 3.29, p = 0.003) remained significant predictors of HWA.

**Conclusion:**

Hypoferritinemia without anemia was prevalent among participants, with lethargy as the primary symptom. Residing in a rural area, being unemployed, use of medication, prolonged menstrual days, and poor dietary intake all significantly raised the risk of HWA. These results show that vulnerable groups need more targeted nutritional education, reproductive health awareness, and better access to health care.

## INTRODUCTION

Iron deficiency is one of the most common public health problems globally, as it affects people of all ages and social classes. According to World Health Organization (WHO) around one billion people have iron deficiency anemia (IDA) and more than two billion people have signs and symptoms of iron deficiency even when their hemoglobin levels are normal.^1^ This condition is known as Hypoferritinemia without anemia (HWA) or iron deficiency without anemia (IDWA). HWA is a condition that is at least twice as common as IDA especially in women of reproductive age but is still not diagnosed or treated, especially in low- and middle-income countries.^2^

Several worldwide studies have proven that patients with HWA develop symptoms like lethargy / feeling of tiredness, inefficient work performance, inadequate focus and absent mindedness, sore tongue, damaged skin, brittle hair including hair loss, fragile nails, prolonged wound healing, rapid heartbeat and restless legs. Such nonspecific symptoms are usually attributed to other causes and treated symptomatically, instead of being appropriately investigated for iron deficiency.^3^ A study from Lebanon cited a 57.5% incidence of HWA among women with heavy menstrual blood loss and fatigue.^4^ Several studies from around the world have also found that women with HWA often have fatigue, hair loss, dizziness, poor condition of hair, skin or nails, psychological and physical symptoms, restless legs, cold intolerance, despite the normal hemoglobin levels.^5^,^6^

In Pakistan, iron deficiency is a well-established public health issue. National nutrition surveys and small-scale research have largely concentrated on IDA using hemoglobin as the critical criterion.^7^ No large- scale studies in Pakistan have addressed concerns on HWA, in contrast to the concerns on IDA, even in the presence of socio-demographic, biological and cultural risk factors like excessive menstruation, high parity, low dietary diversity and poor dietary access for preventive care. These factors make Pakistani women particularly vulnerable to iron depletion. While internationally clinical significance of HWA is being increasingly recognized^8^, a pivotal local and regional gap in studies on HWA still exists in Pakistan, wherein diagnostics mainly rest on anemia-based markers. Thus, women with normal hemoglobin and low iron stores go unnoticed and get relieved of symptoms like fatigue or cognitive deficits by virtue of medication with antidepressants or analgesics.^9^ There is also no published data available for the frequency, risk factors, or response to treatment of HWA in Pakistani women, creating a considerable gap in evidence-based practice for this vulnerable population.

The current research seeks to evaluate the frequency and determinants of HWA among reproductive-age women in Lahore, Pakistan. The purpose of this cross-sectional analytical study design is to examine the association of HWA with socio-demographic, biological and dietary determinants. By recognizing at-risk populations and assessing clinical outcomes, this research aims to facilitate early detection and treatment of HWA. This would eventually lead to better diagnoses and evidence-based practices that help healthcare professionals manage patients better and help improve the quality of life for women in low-resource settings.

## METHODOLOGY

A list of government and private hospitals was obtained from the healthcare department in Lahore, Pakistan. From the list on the basis of highest number of beds, two government hospitals and two private hospitals were selected namely Lahore General Hospital and Sir Ganga Ram Hospital and from private sector Gulab Devi Hospital and Fatima Memorial Hospital along with their adjoining areas. Data was gathered from the medical outpatient departments (OPDs), visited by women for routine consultations and non-specialized healthcare services. The term “adjoining areas” refers to the nearby communities and neighborhoods that are within the hospitals’ catchment areas such as women’s hostels and Quran centers.

### Sample size:

Using a 1.96 z-score (for 95% confidence interval), 0.41 as *p*, 0.59 as *q* (1-*p*), and 0.06 as the margin of error, the minimum sample size calculated was 259.^10^ The sample size was increased four times to 1036 due to the selection of four study sites for data collection. To accommodate a 30% non-response rate 11,12, the final sample size was increased to 337 each site, resulting in a combined total of 1,347 participants for the cross-sectional study design.

### Sampling technique:

A simple random sampling method was used to make sure that each eligible participant had the same chance of being chosen to reduce selection bias

### Sample Selection:

Women were included if they were in reproductive age of 18 to 45 years with any of the symptom mentioned in the inclusion criteria indicative of iron deficiency. The eligibility criteria was accessed through the study questionnaire to ensure precise comprehension and reporting by each participant.

### Inclusion Criteria:

- Females of reproductive age i.e., 18 to 45 years.
- Having at least any one of the listed clinical symptoms of iron deficiency: lethargy/ feeling of tiredness, inefficient work performance, inadequate focus and absent mindedness, sore tongue, damaged skin, brittle hair including hair loss, fragile nails, prolonged wound healing, rapid heartbeat and restless legs.

### Exclusion Criteria:

- Premature menopause.
- History of iron supplements, multivitamins and blood transfusion during the last three months.
- Pregnant or lactating females.
- Any history of blood disease like Hemophilia or thalassemia.
- Unable to give medical history.
- Any mental illness.

### Data collection procedure:

### Informed consent:

All participants were provided with written informed consent after a thorough explanation of the study purpose, procedures, potential risk and benefits, thereby ensuring voluntary participation and confidentiality in compliance with ethical standards.

### Data collection method:

Data were collected via simple random sampling from medical outpatient departments in four hospitals, accompanied by recruitment through medical camps organized in women hostels and Quran centers to include community-based women. After getting informed consent, trained interviewers conducted face to face interviews, and a phlebotomist collected blood samples for serum ferritin and CBC analysis.

### Ethical approval:

The ethical approval was obtained from the ethical review committee of University of Lahore, Pakistan vide Ref no: REC-UOL-248-10-2022 dated October, 13th, 2022. The study strictly followed ethical guidelines for conducting epidemiological studies. This cross-sectional study is the part of the dissertation project of the first author on the topic of “Hypoferritinemia without anemia among reproductive age females: Prevalence, determinants and treatment outcomes”.

### Data collection tool:

The questionnaire used was divided into three sections.

### Section 1: Qualifying questions:

Included respondents age, history of menopause, blood transfusion, iron supplementation in last three months and history of symptoms.

### Section 2: Sociodemographic variables:

Consisted of level of education, region, religion, monthly family income, marital status, if married, number of children and working status.

### Section 3: Determinants:

Included medication history, number of days of menstrual bleeding, number of pads changed each day, frequency of stools, family history of anemia, history of comorbidities, frequency of meals, skipping of breakfast, lunch and dinner and dietary diversity.

### Operationalization of variables:

*Section 1* included qualifying questions to determine eligibility for inclusion. Age was recorded in completed years and later coded as 18-25, 26-35 and 36-45. Other qualifying questions on premature menopause, history of blood transfusion and history of iron supplementation in last three months and symptoms of iron deficiency were accessed dichotomous (Yes/No).

*Section 2* covered the participant’s socio-demographics. Level of education was categorized as no formal education, primary, middle, secondary, graduation, and higher graduation. The region was classified as either urban or rural. Monthly income was divided into four categories: <20,000 PKR, 20,000-50,000 PKR, 50,000-100,000 PKR, and >100,000 PKR. The number of children was coded into 0, 1-2, and ≥3, and the marital status was noted as never married and ever married. Employment status included ever employed and never employed.

*Section 3* evaluated the determinants pertaining to biological and dietary determinants. Use of medication during the previous three months was noted dichotomously (yes/no). The number of days of menstrual bleeding, classified as either normal (1-7 days) or prolonged (>7 days), and the quantity of sanitary pads used daily, coded as ≤2 or >2 pads. The frequency of bowel movements was categorized as either normal (occurring 1-2 times daily) or altered (occurring more frequently). Anemia in the family history was recorded dichotomously (yes/no). Comorbidities were also assessed using a dichotomous approach (yes/no). Meal frequency (<2 meals or >3 meals) and meal-skipping behavior for breakfast, lunch, and dinner were used to assess dietary habits. These behaviors were classified as occasionally, once to twice per week, three to four, or more than four times per week. After being evaluated on a 24-hours recall, dietary diversity was recoded as low (≤5 food groups) and moderate (6-10 food groups).

### Statistical analysis:

Data was analyzed using SPSS version 24. For the descriptive analysis, univariate analysis including frequencies and percentages were used, while mean and standard deviation (in case of normally distributed variables) and median (in case of skewed variables) were given for quantitative variables. For inferential statistics, chi-square and binary logistic regression with 95% confidence interval and p-value <0.05 was used. The variables significant at binary logistic regression analysis were placed in multivariate logistic regression analysis. Odds ratios (OR), adjusted odds ratios (AOR), and 95% confidence intervals (CI) are shown. In multivariable logistic regression, all independent variables that were significant at 0.05 level were entered in the model.

## RESULTS

Out of 1347 participants, (29.6%) had HWA (Hb > 12 g/dL, Fe < 30ng/mL), (30.1%) were normal (Hb > 12 g/dL, Fe > 30 ng/mL), (3.49%) had hyperferritinemia (SF >120ng/ml), (24.6%) had iron deficiency anemia (Hb < 12 g/dL, Fe < 30 ng/mL) and (12.2%) had non-iron deficiency anemia (Hb < 12g/dL, Fe >30 ng/mL). The mean age was 29.06 ± 8.942. The mean hemoglobin level was 12.01 ± 1.33. The median serum ferritin was 28.0. A subgroup analysis based on medication history indicated that participants without a history of medication use (n = 1,089) exhibited a mean serum ferritin level of 36.7 ng/mL (range: 4.3-277.9 ng/mL), while those reporting medication use (n = 258) demonstrated a mean level of 42.6 ng/mL (range: 4.5-307.5 ng/mL). Ferritin levels were slightly elevated among medication users; however, this difference was not statistically significant (p = 0.081).

The socio-demographic characteristics of 1,347 study participants is presented in Table I. The mean age was 29.06 ± 8.94 years, and almost half (46.9%) were between 18- 25 years old. The majority of participants were urban residents (80.3%) and muslim (90.9%). Regarding education, 32.5% had more than 13 years of school, and (5.3%) had no formal schooling. (52.3%) of them had been married before, and (46.8%) of them had 3-4 children.

**Table-I:** Frequency distribution of socio-demographic characteristics of study participants (n=1347).

Variable	Frequency (n)	Percentage %
**Age in years (n = 1347)**		
18-25	632	46.9
26-35	351	26.1
36-45	364	27.0
**Mean + SD: 29.06 + 8.942**		
**Highest level of education (n = 1347)**		
No Education	72	5.3
Primary (1-5)	243	18.0
Middle (6-10)	282	20.9
Secondary (11-12)	312	23.2
Higher (13 and above)	438	32.5
**Region (n = 1347)**		
Rural	266	19.7
Urban	1081	80.3
**Religion (n = 1347)**		
Islam	1225	90.9
Christianity	122	9.1
**Monthly income (PKR) ^b^ (n = 1347)**		
< 20,000	31	2.3
20,001-50,000	474	35.2
50,001-100,000	558	41.4
Above 100,000	284	21.1
**Marital status ^a^ (n = 1347)**		
Never Married	642	47.7
Ever Married	705	52.3
**If married, no. of children (n=705)**		
0	66	4.9
1-2	212	30.1
3-4	330	46.8
5 & above	97	13.8
**Working status (n= 1347) ^c^**		
Currently employed	373	27.7
Currently not employed	974	72.3

a Includes single, married, widow and divorced.

b 1 USD = 278 PKR.

c Includes unemployed, business, employed, house-wives and students.

The frequency distribution of self-reported symptoms of IDA among 1347 participants is presented in Table-II. Lethargy (90.7%) was the most common symptom, followed by damaged skin, brittle hair (including hair loss), and fragile nails (65.0%) and restless legs (46.7%). Symptoms like poor work performance (28.3%), lack of focus and absent-mindedness (28.3%), rapid heartbeat (23.6%), and sore tongue (23.2%) were less frequently reported. Prolonged wound healing (3.7%) was the least common. These results show that most participants had at least one symptom of IDA, despite not being clinically anemic.

**Table-II:** Frequency distribution of symptoms of iron deficiency anemia among each participant (n = 1347).

Variables	Yes
	Frequency (%)
Lethargy	1222 (90.7)
Inefficient work performance	381 (28.3)
Inadequate focus & absent mindedness	381 (28.3)
Sore tongue	312 (23.2)
Damaged skin, brittle hair including hair loss, fragile nails	876 (65.0)
Prolonged wound healing	50 (3.7)
Rapid heartbeat	318 (23.6)
Restless legs	629 (46.7)

The association between socio-demographic, biological, and dietary factors and HWA among participants is shown in Table-III. Education level (*p* < 0.001), region (p = 0.003), monthly income (*p* < 0.001), number of children (p = 0.012), and working status (p = 0.015) were statistically associated with HWA. Among biological determinants, history of medication, menstrual duration, number of pads used, stool frequency, family history of anemia, celiac disease, and diabetes exhibited significant associations (*p* < 0.05) with HWA. Females with prolonged menstruation, and a family history of anemia exhibited elevated HWA rates. Dietary factors such as meal frequency, meal omission, and dietary diversity score were significantly correlated (*p* < 0.001). Participants who consume fewer meals, often skipped meals, and had a limited variety of foods were more likely to have anemia.

**Table-III:** Bivariate analysis of sociodemographic, biological, and dietary determinants associated with HWA in Lahore, Pakistan (Chi-Square) (n = 805).

Variable	HWA		Total	p-Value
	No n (%) (n = 406)	Yes n (%) (n = 399)	n (%) (n = 805)
**Socio-demographic variables**				
Age (n = 805)				
18-25	204 (51.4)	193 (48.6)	397 (49.3)	0.293
26-35	103 (46.2)	120 (53.8)	223 (27.7)	
36-45	99 (53.5)	86 (46.5)	185 (23.0)
**Highest level of education (n = 805)**				
No Education	6 (25.0)	18 (75.0)	24 (3.0)	<0.001
Primary	37 (25.9)	106 (74.1)	143 (17.8)	
Middle	65 (40.9)	94 (59.5)	159 (19.8)	
Secondary	124 (60.8)	80 (39.0)	204 (25.3)	
Higher	174 (63.3)	101 (36.7)	275 (34.2)
**Region (n = 805)**				
Urban	340 (53.0)	301 (47.0)	641 (79.6)	0.003
Rural	66 (40.2)	98 (59.8)	164 (20.4)
**Monthly family income in PKR (n = 805)**				
<20,000	6 (33.3)	12 (66.7)	17 (2.1)	<0.001
20,001-50,000	53 (23.3)	174 (77.0)	227 (28.2)	
50,001-100,000	186 (54.9)	153 (45.1)	339 (42.1)	
Above 100,000	162 (73.0)	60 (27.0)	222 (27.6)
**Marital Status (n = 403)**				
Never Married	204 (50.7)	198 (49.3)	402 (49.9)	0.860
Ever Married	202 (50.1)	201 (49.9)	403 (50.1)
**Number of Children (n = 403)**				
0	24 (57.1)	18 (42.9)	42 (10.4)	0.012
1-2	77 (59.2)	55(41.7)	132 (32.8)	
3-4	87 (46.8)	98 (53.0)	185 (45.9)	
5 & above	14 (31.1)	30 (68.2)	44 (10.9)
**Working Status (n = 805)**				
Employed	141 (56.9)	107 (43.1)	248 (30.8)	0.015
Unemployed	265 (47.6)	292 (52.4)	557 (69.2)
**Biological determinants**				
**History of medication (n = 805)**				
No	382 (53.1)	337 (46.9)	719 (89.3)	<0.001
Yes	24 (27.9)	62 (72.1)	86 (10.7)
**No. of days of menstrual bleeding (n = 805)**				
< 7	384 (54.8)	317 (45.2)	701 (87.3)	< 0.001
> 7	22 (21.2)	82 (78.8)	104 (12.7)
**Pads changed per day (n = 805)**				
<2	377 (54.6)	314 (45.4)	691 (85.8)	< 0.001
>2	29 (25.4)	85 (74.6)	114 (14.2)
**Frequency of stools (n = 805)**				
<1	377 (54.2)	319 (45.8)	696 (86.5)	< 0.001
>2	29 (26.6)	80 (73.4)	109 (13.5)
**Family history of anemia (n = 805)**				
No	366 (52.5)	331 (47.5)	697 (86.6)	< 0.001
Yes	40 (37.0)	68 (63.0)	108 (13.4)
**Celiac disease (n = 805)**				
No	399 (51.2)	381 (48.8)	780 (96.9)	0.023
Yes	07 (0.9)	18 (72.0)	25 (3.1)
**Diabetes (n = 805)**				
No	394 (51.2)	375 (48.8)	769 (95.5)	0.036
Yes	12 (33.3)	24 (66.7)	36 (4.5)
**Dietary determinants**				
**Frequency of meals (n = 805)**				
< 2	109 (28.2)	277 (71.8)	386 (48.0)	< 0.001
> 3	297 (70.9)	122 (29.1)	419 (52.0)
**Meal Skipping Skipping Breakfast (n = 805)**				
Occasionally	337 (56.4)	260 (43.6)	597 (74.2)	< 0.001
1-2 times	30 (40.0)	45 (60.0)	75 (9.3)	
3-4 times	21 (26.6)	58 (73.4)	79 (9.8)	
>4 times	18 (33.3)	36 (66.7)	54 (6.7)
**Skipping Lunch (n = 805)**				
Occasionally	340 (56.1)	266 (44.0)	606 (75.3)	< 0.001
1-2 times	30 (33.3)	60 (65.9)	90 (11.2)	
3-4 times	27 (32.9)	55 (67.1)	82 (10.2)	
>4 times	9 (33.3)	18 (66.7)	27 (3.4)
**Skipping Dinner (n = 805)**				
Occasionally	366 (54.1)	311 (46.0)	677 (84.1)	< 0.001
1-2 times	21 (37.8)	36 (62.1)	57 (7.1)	
3-4 times	11 (25.6)	32 (74.4)	43 (5.3)	
>4 times	8 (28.6)	20 (71.4)	28 (3.5)
**Dietary diversity score (n = 805)**				
Low	53 (26.4)	148 (73.6)	201 (25.0)	< 0.001
Moderate	353 (58.4)	251 (41.6)	604 (75.0)
p-Values have been calculated using chi-square test				

The results of simple binary logistic regression are presented in Table IV. Individuals with secondary (OR = 0.22; 95% CI: 0.08-0.57; p = 0.002) and higher education (OR = 0.19; 95% CI: 0.07-0.50; p = 0.001) exhibited lower odds of HWA in comparison to those with less education. Individuals from rural areas (OR = 1.68; 95% CI: 1.18-2.38; p = 0.004), possessing lower income (<50,000 PKR), and those who are unemployed (OR = 1.45; 95% CI: 1.07-1.96; p = 0.015) exhibited a higher likelihood to have HWA. A history of medication use (OR = 2.93; 95% CI: 1.79-4.80; p = 0.000), >7 days of menstrual bleeding (OR = 4.59; 95% CI: 2.80-7.51; p = 0.000), changing >2 pads per day (OR = 3.52; 95% CI: 2.25-5.50; p = 0.000), frequent stools (more than 2/day) (OR = 3.26; 95% CI: 2.08-5.11; p = 0.000), a family history of anemia (OR = 1.88; 95% CI: 1.24-2.86; p = 0.003), celiac disease (OR = 2.69; 95% CI: 1.11-6.52; p = 0.028), diabetes (OR = 2.10; 95% CI: 1.04-4.26; p = 0.040), consumed <2 meals per day (OR = 6.19; 95% CI: 4.56-8.40; p = 0.000), skipped breakfast (1-2 times/week: OR = 1.94; 95% CI: 1.19-3.17; p = 0.008; 3-4 times/week: OR = 3.58; 95% CI: 2.12-6.05; p = 0.000), lunch (1-2 times/week: OR = 2.56; 95% CI: 1.60-4.08; p = 0.000), and dinner (3-4 times/week: OR = 3.42; 95% CI: 1.70-6.91; p = 0.001), and exhibited a low dietary diversity score (OR = 3.93; 95% CI: 2.76-5.59; p = 0.000) were all significantly linked to HWA.

**Table IV:** Binary and multivariable logistic regression for factors associated with HWA in Lahore, Pakistan (n = 805).

Variable	OR	95% CI	p-Value	AOR	95% CI	p-Value
**Highest level of education (n = 805)**						
No Education	1			1		
Primary	0.955	(0.352-2.588)	0.928	0.499	(0.125-1.988)	0.325
Middle	0.482	(0.182-1.280)	0.143	0.633	(0.161-2.488)	0.512
Secondary	0.215	(0.082-0.565)	0.002	0.298	(0.073-1.226)	0.094
Higher	0.193	(0.074-0.503)	0.001	0.831	(0.158-4.375)	0.828
**Region (n =805)**						
Urban	1			1		
Rural	1.677	(1.184-2.377)	0.004	3.036	(1.229-7.503)	0.016
**Monthly family income in PKR^b^ (n = 805)**						
<20,000	1			1		
20,001-50,000	6.480	(2.191-19.168)	0.001	3.368	(0.522-21.734)	0.202
50,001-100,000	8.864	(5.784-13.585)	0.000	11.292	(3.891-32.768)	0.000
Above 100,000	2.221	(1.541-3.201)	0.000	3.702	(1.496-9.159)	0.005
**Number of Children (n = 403)**						
0	1			1		
1-2	0.952	(0.472-1.922)	0.892	0.879	(0.280-2.760)	0.825
3-4	1.502	(0.764-2.952)	0.238	0.643	(0.211-1.964)	0.438
5 & above	2.857	(1.184-6.894)	0.019	2.401	(0.585-9.849)	0.224
**Working status (n = 805)**						
Employed	1			1		
Unemployed	1.452	(1.074-1.963)	0.015	3.541	(1.483-8.455)	0.004
**History of medication (n = 805)**						
No	1			1		
Yes	2.928	(1.788-4.796)	0.000	15.016	(4.610-48.914)	0.000
**No. of days of menstrual bleeding (n = 805)**						
< 7	1			1		
> 7	4.585	(2.800-7.506)	0.000	7.488	(2.744-20.436)	0.000
**Pads changed per day (n = 805)**						
<2	1			1		
>2	3.519	(2.250-5.504)	0.000	3.209	(1.054-9.774)	0.040
**Frequency of stools (n = 805)**						
<1	1			1		
>2	3.260	(2.078-5.114)	0.000	1.743	(0.648-4.691)	0.271
**Family history of anemia (n = 805)**						
No	1			1		
Yes	1.880	(1.238-2.855)	0.003	2.239	(0.903-5.556)	0.082
**Celiac disease (n = 805)**						
No	1			1		
Yes	2.693	(1.112-6.520)	0.028	2.479	(0.320-19.196)	0.385
**Diabetes (n = 805)**						
No	1			1		
Yes	2.101	(1.036-4.262)	0.040	0.330	(0.081-1.347)	0.122
**Frequency of meals (n = 805)**						
> 3	1			1		
< 2	6.187	(4.556-8.400)	0.000	9.448	(2.885-30.939)	0.000
**Meal Skipping Skipping Breakfast (n = 805)**						
Occasionally	1			1		
1-2 times	1.944	(1.192-3.172)	0.008	4.670	(1.173-18.591)	0.029
3-4 times	3.580	(2.118-6.050)	0.000	2.901	(0.535-15.712)	0.217
>4 times	2.592	(1.439-4.669)	0.002	0.250	(0.045-1.389)	0.113
**Skipping Lunch (n = 805)**						
Occasionally	1			1		
1-2 times	2.556	(1.603-4.077)	0.000	0.599	(0.170-2.114)	0.426
3-4 times	2.604	(1.599-4.240)	0.000	0.856	(0.230-3.187)	0.817
>4 times	2.556	(1.130-5.782)	0.024	3.299	(0.407-26.769)	0.264
**Skipping Dinner (n = 805)**						
Occasionally	1			1		
1-2 times	2.017	(1.154-3.528)	0.014	1.203	(0.302-4.794)	0.793
3-4 times	3.424	(1.698-6.905)	0.001	1.170	(0.216-6.345)	0.856
>4 times	2.942	(1.278-6.772)	0.011	0.831	(0.122-5.668)	0.850
**Dietary diversity score (n = 805)**						
Moderate	1			1		
Low	3.927	(2.759-5.590)	0.000	3.290	(1.517-7.137)	0.003

***Abbreviation:*** 1 is the reference category; OR, odds ratio; AOR, adjusted odds ratio, CI; confidence interval.

After adjusting, rural region (AOR = 3.04; 95% CI: 1.23-7.50; p = 0.016), income between 50,001-100,000 PKR (AOR = 11.29; 95% CI: 3.89-32.77; p = 0.000), unemployment (AOR = 3.54; 95% CI: 1.48-8.46; p = 0.004), history of medication (AOR = 15.02; 95% CI: 4.61-48.91; p = 0.000), >7 days of menstrual bleeding (AOR = 7.49; 95% CI: 2.74-20.44; p = 0.000), >2 pads per day (AOR = 3.21; 95% CI: 1.05-9.77; p = 0.040), <2 meals/day (AOR = 9.45; 95% CI: 2.89-30.94; p = 0.000), skipping breakfast 1-2 times/week (AOR = 4.67; 95% CI: 1.17-18.59; p = 0.029), and low dietary diversity (AOR = 3.29; 95% CI: 1.52-7.14; p = 0.003) remained significantly associated with HWA.

## DISCUSSION

The most effective approach for reducing the burden of iron-related morbidity is the early identification of iron deficiency, accompanied by timely dietary and clinical interventions. In numerous low- and middle-income countries, ferritin testing is uncommon, and anemia screening depends solely on hemoglobin, permitting subclinical deficiency to go unnoticed. Our findings emphasize the necessity of incorporating ferritin-based assessment into standard women health services.

This is the first hospital and community-based study from Lahore that investigates the determinants of hypoferritinemia without anemia (HWA) among reproductive-age women. Around one-third had HWA despite their hemoglobin levels being normal. This prevalence aligns with findings from India, Lebanon, and Iran, where the rates of HWA among women of childbearing age vary from 30% to 57%.^4^,^5^ Most of the people who took part lived in cities, but women who lived in rural areas were much more likely to have HWA.

Lethargy, hair loss, and restless legs were the predominant symptoms observed in our study, which mirrors findings from Iran^5^ and India,^8^ where non- anemic women with low ferritin levels reported fatigue and compromised hair and skin health. Similarly, in the research conducted by Sawada et al., demonstrated that iron deficiency without anemia can yield clinically significant symptoms of anger and fatigue.^13^ This emphasizes the significance of serum ferritin evaluation, as dependence solely on hemoglobin testing may lead to the under diagnosis of early iron deficiency.

Living in a rural area, being unemployed, and use of medication were all linked to a higher risk of HWA. Menstrual bleeding lasting more than seven days and using more than two sanitary pads per day remained strong predictors. Eating habits were also strongly linked to HWA included skipping breakfast, eating less than two meals a day, and having low dietary diversity. These correlations correspond with findings from South Asia and other resource-limited settings indicating that geographic disadvantage, economic instability, significant menstrual blood loss, and insufficient or monotonous diets lead to HWA.

There are plausible reasons for these patterns. Living in a rural area often coincides with limited primary care, fewer fortified foods, and low nutritional literacy.^14^ Unemployment was also strongly linked to HWA, which shows how economic instability can lead to nutritional deficiencies. Being financially low makes it difficult to consume iron-rich foods and get medical care, which makes iron status worse. These results align with research conducted in Kenya and Canada, which indicated that unemployed or low-income women exhibited a greater prevalence of iron deficiency and a diminished probability of undergoing ferritin level screening.^15^,^16^ These observations highlight the convergence of economic vulnerability and nutritional health, underscoring the necessity for equitable access to dietary and medical resources showing that socioeconomic status is also a significant factor affecting HWA.

History of medication use was another important determinant that was linked to HWA. Long-term use of medications may reflect health problems like diabetes, gastrointestinal disorders, or infections that impair the body’s ability to absorb or utilize iron.^17^,^18^ This outcome is consistent with prior research indicating a correlation among chronic illness, polypharmacy, and iron deficiency. In this context, healthcare professionals should regard medication history as a significant criterion when evaluating women susceptible to iron deficiency.

Factors related to reproductive health also played a major role. Women who had menstrual bleeding that lasted longer than seven days or who used more than two sanitary pads a day were much more likely to have HWA. This finding aligns with research conducted in Iran, Thailand, and the United Kingdom, where menstrual blood loss was a significant indicator of iron deficiency.^8^,^18^-^20^ Another study from Sweden found that women with heavy menstrual bleeding had lower quality of life scores, which shows how chronic iron loss can affect daily life.^21^ A significant contribution of this study is the utilization of the quantity of sanitary pads as a proxy indicator for menstrual blood loss an effective and culturally pertinent method for evaluating menstrual patterns in population-based research.

Dietary behavior emerged as a significant determinant of HWA. Over one-third (n=147) of women with HWA exhibited a low dietary diversity score (1-5 food groups), whereas women with normal ferritin levels demonstrated a moderate score (6-10 food groups). Women who consume less than two meals a day, skipped breakfast, and had a limited dietary diversity were at higher risk. Similar results were found in studies from Nigeria and Pakistan, where low dietary diversity scores were linked to not affording nutrient rich foods by virtue of being economically challenged.^22^,^23^ Furthermore, it was found that women who often missed breakfast or lunch and consumed less animal protein, fruits, and vegetables were more likely to have low ferritin levels. Research shows that individual DDS is a good measure of nutrient adequacy and is closely related to socioeconomic status and food security of the household.^24^

In countries like Pakistan cultural implications also play a major role where women often rely on leftover food. This is partly influenced by traditional household practices that may limit women’s dietary diversity and intake of iron rich food.^22^ In contrast, a study in Japan found that men generally consume more total energy, carbohydrates and nutrients than women (p < 0.05), pointing out gender-based differences in wealthier countries also.^25^

This study offers significant baseline evidence regarding the frequency and determinants of HWA in women of reproductive age; however, additional research is necessary to expand upon these findings. Longitudinal studies are necessary to ascertain causal relationships between identified determinants and ferritin depletion over time. Interventional studies evaluating the efficacy of iron supplementation, menstrual health education and dietary counseling may contribute to the formulation of targeted prevention strategies. Furthermore, investigating the molecular and inflammatory mechanisms that contribute to HWA would yield a more profound understanding of its pathophysiology and its enduring effects on women’s health.

### Strengths and limitations:

The large sample size (n = 1,347) and the inclusion of a variety of variables, ranging from medical history and menstrual characteristics to socio-demographics and dietary patterns, are among the study’s main advantages. This kind of multi-faceted analysis gives a clearer picture of what leads to HWA in real-life situations, especially in individuals with limited resources. However, the study has few limitations. First, the cross-sectional design precludes causality. It is not possible to establish causal relationships between the identified determinants and HWA; the observed associations suggest association rather than causation. Second, history of medication, comorbidities, dietary diversity pattern and symptoms were self-reported which could lead to recall and reporting bias. Third, participants may be more likely to show social desirability bias or exaggerate their symptoms to get benefits or participate in the study, which could affect the accuracy and reliability of our results. Finally, serum ferritin levels can be falsely high when there is inflammation, even though medication use and comorbidities were taken into account in the questionnaire.

## CONCLUSION

This study demonstrates that HWA is a significant and underrecognized health concern among reproductive-age women in Pakistan, presenting with diverse clinical symptoms influenced by sociodemographic disadvantages, biological determinants and poor dietary habits. While current practice often includes hemoglobin testing and iron studies for women with identifiable risk factors, our findings emphasize that hemoglobin alone is an insufficient marker of iron status. To accurately detect iron deficiency in its early stages, serum ferritin levels must also be evaluated. Incorporating ferritin testing alongside a comprehensive clinical history, especially in symptomatic women or those with known risk factors, can significantly improve early detection and timely intervention. These results highlight the urgent need to include ferritin assessment, menstrual health education and targeted nutritional programs into national health policies. Such measures are essential to prevent the progression to iron deficiency anemia and improve women’s overall health, cognitive performance and reproductive outcomes.

### Authors’ contribution:

**SJK:** Conceived, designed, collected data and did statistical analysis, writing, editing of manuscript, is responsible for integrity of research

**AMA & RZ:** Critical review, literature search, final approval of manuscript.
